# Could the Systemic Inflammatory Response Index be a Marker for the Non-Dipper Pattern in Newly Diagnosed Hypertensive Patients?

**DOI:** 10.1007/s12012-025-09977-3

**Published:** 2025-02-24

**Authors:** Mustafa Kaplangoray, Kenan Toprak, Cuneyt Caglayan, Edhem Deveci, Enes Celik, Umut Uyan, Cihan Aydın

**Affiliations:** 1 Cardiology Department, Acıbadem Eskişehir Hospital, Eskişehir, Turkey; 2 Department of Cardiology, Faculty of Medicine, Harran University, Şanlıurfa, Turkey; 3 Department of Medical Biochemistry, Faculty of Medicine, Bilecik Şeyh Edebali University, Bilecik, Turkey; 4 Department of Cardiology, University of Health Sciences Mehmet Akif İnan Research and Training Hospital, Sanlıurfa, Turkey; 5 Department of Cardiology, Republic of Turkey Ministry of Health Ödemiş State Hospital, İzmir, Turkey; 6 Department of Cardiology, Tekirdag Namık Kemal University, Tekirdağ, Turkey

**Keywords:** Systemic inflammatory response index, Hypertension, Inflammatory, Non-dipper

## Abstract

Systemic inflammatory response index (SIRI), is associated with prognosis in coronary artery disease (CAD), heart failure (HF), and acute myocardial infarction. This study investigated the relationship between SIRI and non-dipper hypertension. The study retrospectively included a total of 254 naive, newly diagnosed hypertensive individuals based on ambulatory blood pressure monitoring (ABPM), containing 166 dippers (DHT) and 88 non-dippers (NDHT). The SIRI value of all patients was calculated based on neutrophil, monocyte, and lymphocyte counts. The average age of study population was 50.7 ± 9.4 years old, and the male ratio was 68.5%. Compared with DHT, patients in the NDHT group had higher SIRI, monocyte to HDL-C ratio (MHR), neutrophil–lymphocyte ratio (NLR), platelet-lymphocyte ratio (PLR), C-reactive protein (CRP), and neutrophil count, while high-density lipoprotein cholesterol (HDL-C) and lymphocyte count were lower (p < 0.05). The left ventricular mass index (LVMI) was higher in the NDHT group (p < 0.05). Multivariate logistic regression analysis showed that SIRI, LVMI, and HDL-C were independent predictor factors for NDHT. ROC curve analysis determined the optimal SIRI cut-off value for predicting NDHT diagnosis to be 2.41 (sensitivity 69.3%, specificity 64.5%, area under the receiver operating characteristic curve, 0.743; *p* < 0.001). The AUC values obtained for SIRI, MHR, NLR, PLR, HDL-C, and LVMI parameters in the ROC curve analysis were compared pairwise. The results demonstrated that SIRI’s discriminative capacity in predicting NDHT was superior to all other indices. SIRI is an independent and significant predictor factor for NDHT and is superior in predicting NDHT diagnosis compared with HDL-C, MHR, LVMI, NLR, and PLR.

**Graphical Abstract:**

Relationship between SIRI and non-dipper hypertension. Can inflammation-based hematological parameters serve as an alternative to ambulatory blood pressure monitoring in the diagnosis of non-dipper hypertension? *LAd* Left Atrium Diameter; *HDL-C* High-Density Lipoprotein Cholesterol; *LDL-C* Low-Density Lipoprotein Cholesterol; *NLR* Neutrophil/Lymphocyte Ratio; *PLR* Platelet/Lymphocyte Ratio; *SIRI* Systemic İnflammatory Response İndex; *LVMI* Left Ventricular Mass İndex; *MHR* Monocyte To Hdl-C Ratio

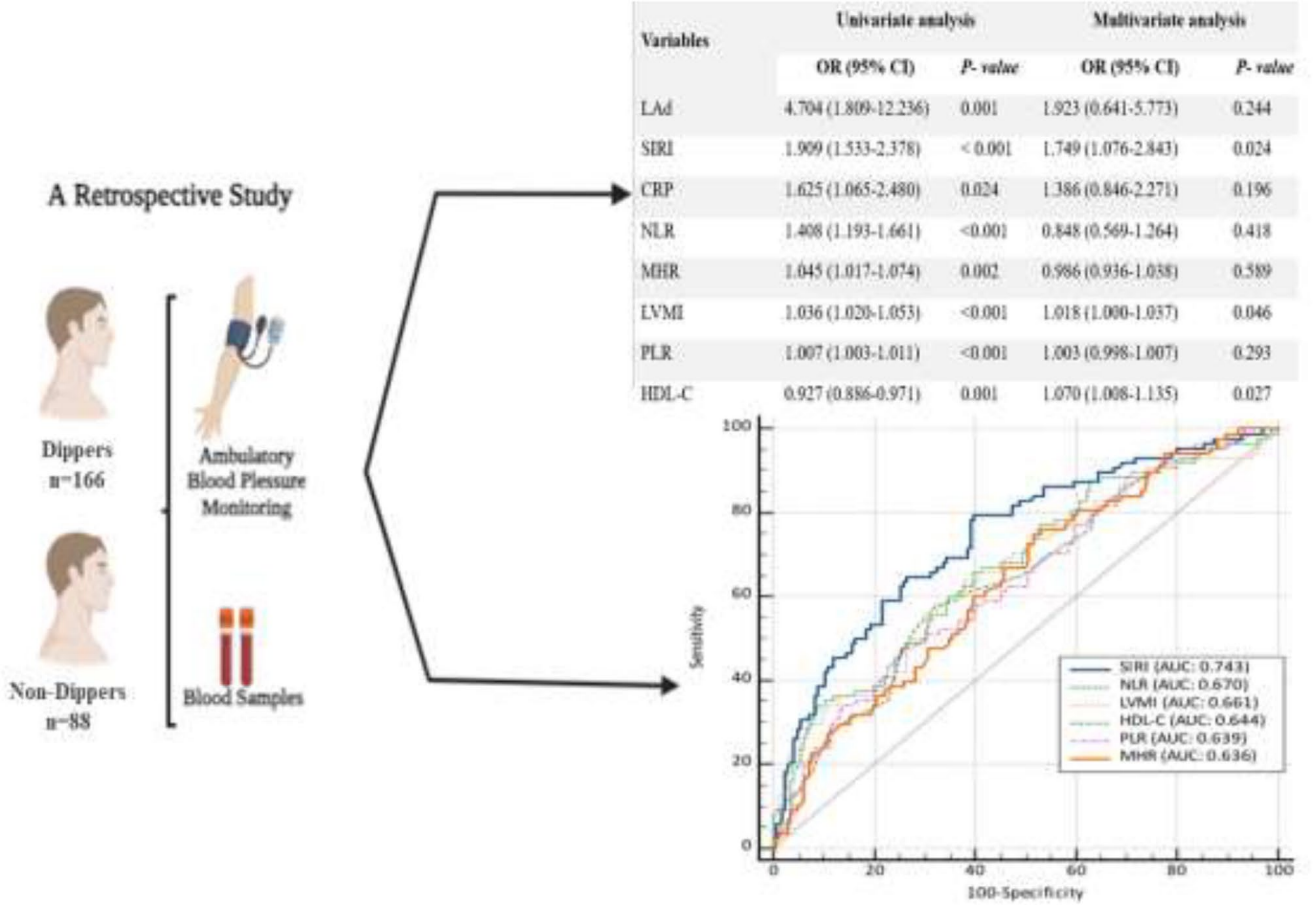

## Introduction

Hypertension alone is responsible for 10.4 million deaths globally each year. In addition to being a major risk factor for cardiovascular diseases, uncontrolled hypertension has been reported to be associated with higher rates of morbidity and death [1]. Ambulatory blood pressure monitoring (ABPM) is more effective than cuff measurement in determining the cardiovascular events risk in hypertensive patients [2]. Significant circadian variations in blood pressure (BP) can be detected with ABPM measurements. A decrease of 10% or more in nighttime BP values compared with daytime values defines dipper hypertension (DHT), whereas a decrease of less than 10% defines non-dipper hypertension (NDHT) [3]. Several studies have shown that NDHT is associated with increased cardiovascular diseases and target organ damage compared with DHT [4]. Performing ABPM on every hypertensive patient might not be practical to in practice due to its cost and accessibility concerns. Consequently, studies on low-cost, readily available markers for NDHT diagnosis have been crucial.

The relationship between inflammation and hypertension is well-established, and this relationship has been supported by several recent studies. Furthermore, a number of studies have revealed that, patients with NDHT have higher levels of inflammatory markers compared with those with DHT [5–7]. Neutrophil–lymphocyte ratio (NLR), monocyte to HDL-C ratio (MHR) and platelet-lymphocyte ratio (PLR) are parameters calculated using peripheral blood biomarkers such as lymphocytes, neutrophils, and platelets, and their correlation with NDHT has been established. [8, 9]. Recently, systemic inflammatory response index (SIRI), an index calculated by multiplying the number of neutrophils by the number of monocytes and dividing it by the number of lymphocytes, was initially reported to be associated with prognosis in cancer patients. [10]. Following studies have revealed that SIRI is also a good marker for predicting prognosis in coronary artery disease (CAD) [11] and heart failure (HF) [12]. However, to our knowledge, no previous study has investigated the relationship between SIRI and NDHT. This study aims to explore this relationship in patients referred to the cardiology clinic for hypertension diagnosis and subsequently confirmed with ABPM.

## Methods

### Study Population

Between January 2021 and July 2023, a total of 254 outpatients who presented to the cardiology department with complaints of high BP, were diagnosed with hypertension via ABPM and had not yet diagnosed with BP, were retrospectively included in the study. Laboratory, echocardiographic, and basic demographic data of the patients were obtained from hospital records during the same visit. Exclusion criteria for the study included chronic kidney disease (eGFR less than 60 mL/min/1.73 m2 or dialysis), active infection (the presence of active infection findings or sedimentation > 20 mm/h, C-reactive protein > 3 mg/L, white blood cell count > 11 × 10⁹/L), presence of coronary artery disease, hyper and hypothyroidism, anemia (hemoglobin below 12 g/dL for women and 13 g/dL for men as per World Health Organization criteria), pregnancy, electrolyte imbalance, sleep apnea syndrome, and prior use of antihypertensive drugs. Patients whose biochemical and hematological parameters were unavailable at the time of their ABPM appointment were also excluded from the study. Additionally, individuals with coronary artery disease, heart failure, and any known malignancy were not included in the study, considering these conditions could affect the SIRI value. The study was performed in accordance with the Helsinki Declaration and received approval from the Ethics Committee of Bilecik Şeyh Edebali University Faculty of Medicine. Due to its retrospective nature, informed consent was waived.

### Laboratory Analysis

All laboratory results for the patients were obtained from blood samples drawn from the antecubital region after 12 h of fasting. Biochemical analyses were carried out using the Roche Cobas 6000 Modular analytical system (Roche Diagnostic Modular Systems, Tokyo, Japan). Serum C-reactive protein (CRP) levels were measured using the nephelometric method (Siemens BN II system, Germany). Complete blood count (CBC) was measured using an automated blood cell counter (Coulter LH 780 Hematology Analyzer, Beckman Coulter Corp, Hialeah, Florida). The Glomerular Filtration Rate (e-GFR) was calculated according to the Chronic Kidney Disease Epidemiology Collaboration (CKD-EPI) formula. Neutrophil, lymphocyte, and platelet counts obtained from hematological examination results were used to calculate NLR and PLR values.

### Calculation of SIRI

SIRI was calculated according to the following equation given below [13], where N represents neutrophil count, M monocyte count, and L lymphocyte count. Data were collected from complete blood count results. SIRI = N × M/L.

### Echocardiographic Examination

Echocardiographic measurements for the patients were obtained from records made during the same session as their ABPM appointment. Echocardiographic measurements were performed by an experienced cardiologist using a 2.5 MHz phased-array transducer (Philips Epiq 7 device, Andover, MA, USA). The left ventricular ejection fraction (LVEF) was calculated using the modified Simpson’s method. At the end of diastole in the parasternal long axis view, the interventricular septum thickness (IVSd), posterior wall (LVPWd), and left ventricular end-diastolic diameter (LVEDd) were measured. The Left Ventricular Mass Index (LVMI) was calculated using the Devereux formula [14]. Additionally, in the parasternal long axis view, the size of the left atrium (LAd) was measured using M-mode.

### Ambulatory Blood Pressure Measurement

A portable digital recording device was used (Schiller MT-300 BP, Baar, Switzerland) For 24-h ABPM. An appropriately sized cuff that would cover at least one-third of the upper arm was selected for each patient. If the blood pressure difference measured from both arms exceeded 10 mmHg, the cuff was attached to the arm with the higher measurement. Blood pressure and heart rate measurements were taken every 15 min during the day and every 30 min at night over a 24-h period. The period between 22:01 and 06:00 was considered the nighttime period, and the period from 06:01 to 22:00 was considered the daytime period. ABPM analysis was conducted according to the diagnostic criteria recommended by current guidelines [15]. A diagnosis of hypertension was made if the 24-h average systolic blood pressure (SBP) was > 130 mmHg and/or diastolic blood pressure (DBP) was > 80 mmHg, daytime average SBP was > 135 mmHg and/or average DBP was > 85 mmHg, and nighttime average SBP was > 120 mmHg and average DBP was > 70 mmHg. A decrease of more than 10% in SBP and DBP measurements at night compared with daytime measurements defined DHT, and patients without this decrease were defined as having NDHT.

### Comorbidity Definition

In-depth review of patient records yielded information about patients’ comorbidity statuses and medication histories. Diabetes mellitus (DM) was defined as a fasting glucose level > 126 mg/dL, hemoglobin A1c > 6.5%, or use of antidiabetic drugs. Dyslipidemia was diagnosed based on the presence of one of the following four results obtained from blood sample analyses after 12 h of fasting: (1) total cholesterol > 200 mg/dL, (2) low-density lipoprotein cholesterol (LDL-C) > 130 mg/dL, (3) high-density lipoprotein cholesterol (HDL-C) < 40 mg/dL in men and < 50 mg/dL in women, and triglyceride level > 150 mg/dL. Patients’ body mass indices were calculated by dividing weight by the square of height measurement (m^2^).

### Statistical Analysis

Statistical analyses were performed using the Statistical Package for the Social Sciences (SPSS for Windows, version 22.0, IBM Corp., Armonk, NY, U.S., 2016) software package. Continuous variables following a normal distribution were presented as mean ± standard deviation, and those not following a normal distribution were presented as median and interquartile ranges. Categorical variables were presented as percentages and compared using the Chi-square or Fisher’s exact test. The Kolmogorov–Smirnov test was used for the assessment the normality of data. To compare the groups; the Independent-samples *t*-test was applied for continuous variables, while the Mann–Whitney U test was utilized for non-normally distributed parameters. Spearman or Pearson’s correlation coefficient tests were used to demonstrate correlations between variables, depending on the distribution of the data. Multivariate regression analyses were performed to determine the independent predictors of presence of NDHT. Baseline variables with significant significance (p < 0.05) by univariate analysis were included in the multivariate logistic regression analysis. ROC analysis was used to determine the cut-off value of SIRI that could predict NDHT. In addition, the discriminative values of SIRI and other markers on NDHT were attempted to be determined by making pairwise comparisons of the areas under the ROC curves with the DeLong test using Medcalc version 19.6.4 statistical software (MedCalc Software Ltd, Ostend, Belgium). The odds ratios (ORs) were presented with 95% respective confidence intervals (CI). A p-value < 0.05 was considered statistically significant in all analyses.

## Results

The study included a total of 254 patients, with 166 in the DHT group and 88 in the NDHT group. The average age of the patients included in the study was 50.7 ± 9.4 years old, and the male ratio was 68.5%. The clinical and demographic characteristics of both groups were comparable. When comparing the two groups from a laboratory perspective, patients in the NDHT group were found to have higher SIRI, MHR, NLR, PLR, CRP, and neutrophil values, while HDL-C and lymphocyte counts were lower (Table 1).

**Table 1:** Basic demographic, clinical characteristics, and laboratory findings of the study population

Variables	Dipper-group (n = 166)	Non-dipper group(n = 88)	p value
Age, years	49.0 (43.0–56.0)	51.0 (45.3–57.0)	0.221
Sex, male,n (%)	116 (66.7%)	58 (65.9%)	0.517
BMI, kg/m^2^	28.0 (26.0–29.0)	28.8 (26.0–30.0)	0.084
Diabetes Mellitus, n (%)	46 (27.7%)	28 (31.8%)	0.493
Dyslipidemia, n (%)	60 (36.1%)	34 (38.6%)	0.143
Smoking, n (%)	64 (38.5%)	35 (39.7%)	0.101
Glucose (mg/dL)	114.0 (87.8–158.0)	108.5 (89.0–130.0)	0.188
Creatinine, (mg/dL)	0.8 (0.7–0.9)	0.81 (0.72–1.09)	0.876
Triglyceride, (mg/dL)	171.5 (126–215.5)	173.0 (131–220)	0.111
TC, (mg/dL)	196 (178–232)	199 (185–241)	0.149
HDL-C, (mg/dL)	42 (36.0–45.0)	37.0 (34.0–43.0)	< 0.001
LDL-C, (mg/dL)	124 (100.8–147)	123.2 (102.3–138.0)	0.974
e-GFR, (ml/min)	96.0 (89.5–102)	95.0 (85.0–100.5)	0.159
Hemoglobin, (g/dL)	14.0 (13.0–15.2)	13.8 (13.0–14.9)	0.265
Platelet count, (× 10^9^/L)	266.0 (221.0–313.0)	258.7 (225.0–363.0)	0.129
CRP, (mg/dL)	0.74 (0.21–1.03)	0.92 (0.3–1.6)	0.023
WBC, (× 10^9^/L)	8.8 (6.0–11.05)	9.6 (7.1–12.0)	0.079
Lymphocyte, (× 10^9^/L)	2.1 (1.7–2.6)	1.8 (1.3–2.2)	< 0.001
Monocyte, (× 10^9^/L)	0.57 (0.6–0.98)	0.67 (0.67–1.1)	0.060
Neutrophil, (× 10^9^/L)	5.4 (4.1–7.1)	6.1 (4.9–8.2)	0.038
MHR	9.9 (6.4–14.4)	14.3 (7.7–16.9)	0.001
NLR	2.6 (1.8–3.6)	3.4 (2.5–5.0)	< 0.001
PLR	129.9 (96.4–175.4)	170.2 (115.4–219-9)	< 0.001
SIRI	1.9(1.3–2.8)	3.1 (2.2–4.3)	< 0.001
Daytime SBP, mm Hg	149.6 ± 9.9	150.6 ± 11.0	0.447
Nighttime SBP, mm Hg	127.9 ± 10.6	144.1 ± 11.3	< 0.001
Daytime DBP, mm Hg	94.6 ± 7.0	94.5 ± 7.2	0.825
Nighttime DBP, mm Hg	81.6 ± 6.2	90.2 ± 7.0	< 0.001
Decline in SBP, %	14.5 ± 3.4	4.3 ± 3.4	< 0.001
Decline in DBP, %	15.2 ± 4.8	5.9 ± 4.7	< 0.001
LVEF, %	57.1 ± 6.8	58.1 ± 6.6	0.329
IVSd, mm	10.5 ± 1.06	11.5 ± 1.25	< 0.001
LVPWd,mm	10.4 ± 1.31	11.4 ± 1.26	< 0.001
LVDd, mm	44.1 ± 5.1	46.3 ± 5.2	0.011
LVMI	87.2 ± 16.1	98.2 ± 19.5	< 0.001
LAd, mm	37.1 ± 2.8	38.3 ± 2.8	0.001

*BMI* Body Mass Index, *CRP* C-Reactive Protein, *IVSd* Interventricular Septum Thickness at End-Diastole, *LVDd* Left Ventricular Dimension at End-Diastole, *LVPWd* LVposterior Wall Thickness at End-Diastole, *LAd* Left Atrium Diameter, *LVEF* Left Ventricular Ejection Fraction, *eGFR* estimated Glomerular Filtration Rate, *TC* Total Cholesterol, *HDL-C* High-Density Lipoprotein Cholesterol, *LDL-C* Low-Density Lipoprotein Cholesterol, *WBC* White Blood Cell, *NLR* Neutrophil/Lymphocyte Ratio, *PLR* Platelet/Lymphocyte Ratio, *SIRI* Systemic Inflammatory Response Index, *MHR* Monocyte to HDL-C Ratio, *SBP* Systolic Blood Pressure, *DBP* Diastolic Blood Pressure, *LVMI* Left Ventricular Mass Index

An analysis of the ABPM measurements revealed that, patients in the NDHT group exhibited average nighttime SBP and DBP values. As expected, the decrease in average nighttime SBP and DBP compared with daytime measurements was found to be greater in the DHT group (Table 1).

**Fig. 1:**
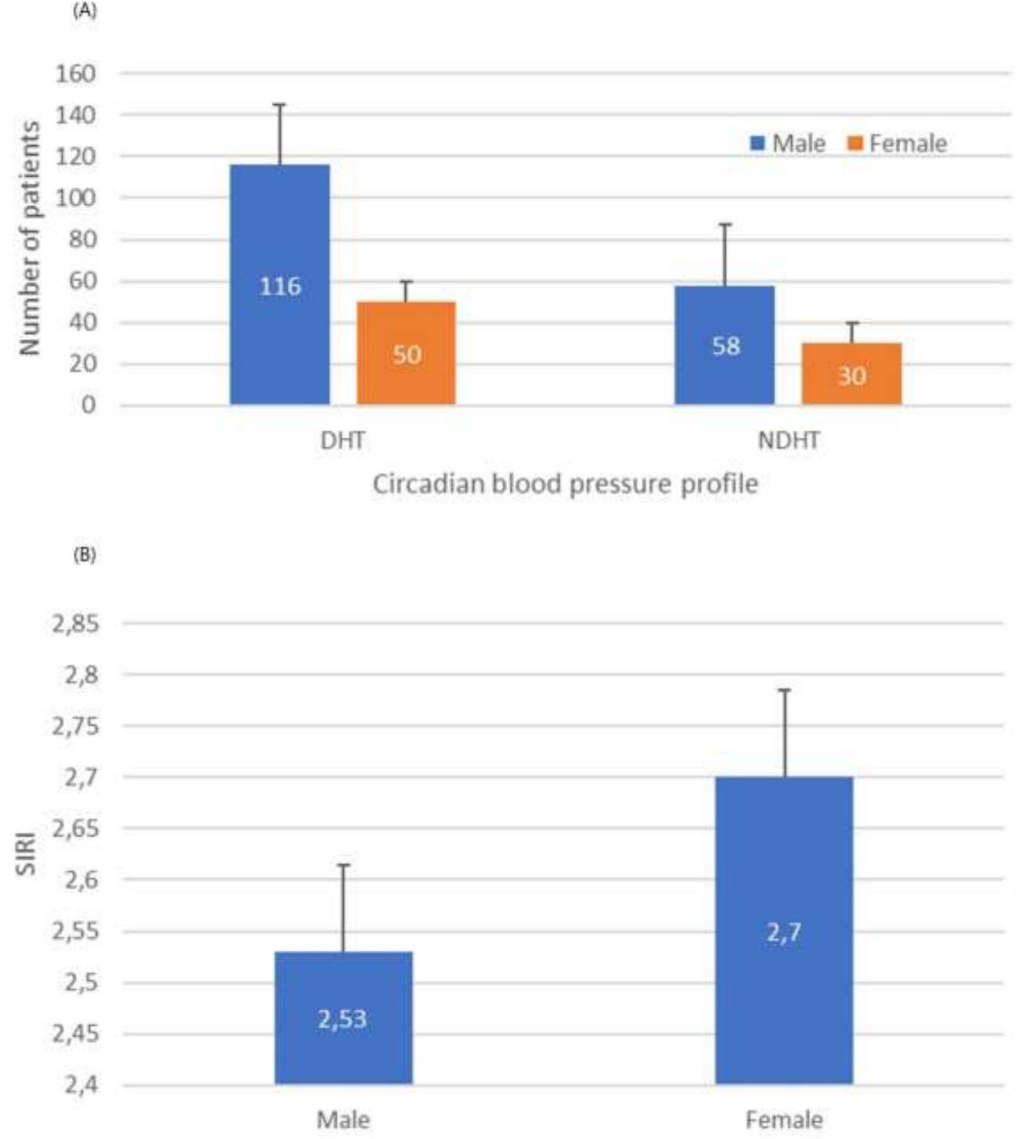
Circadian blood pressure profile and SIRI level according to sex distribution. **A** NDHT rates in male and female included in the study (33.3% and 37.5%, respectively, p = 0.517). **B** Mean SIRI values in male and female included in the study (2.53 and 2.70, respectively, p = 0.387). DHT: Dipper Hypertension, NDHT: Non-Dipper Hypertension, SIRI: Systemic Inflammatory Response Index

**Fig. 2:**
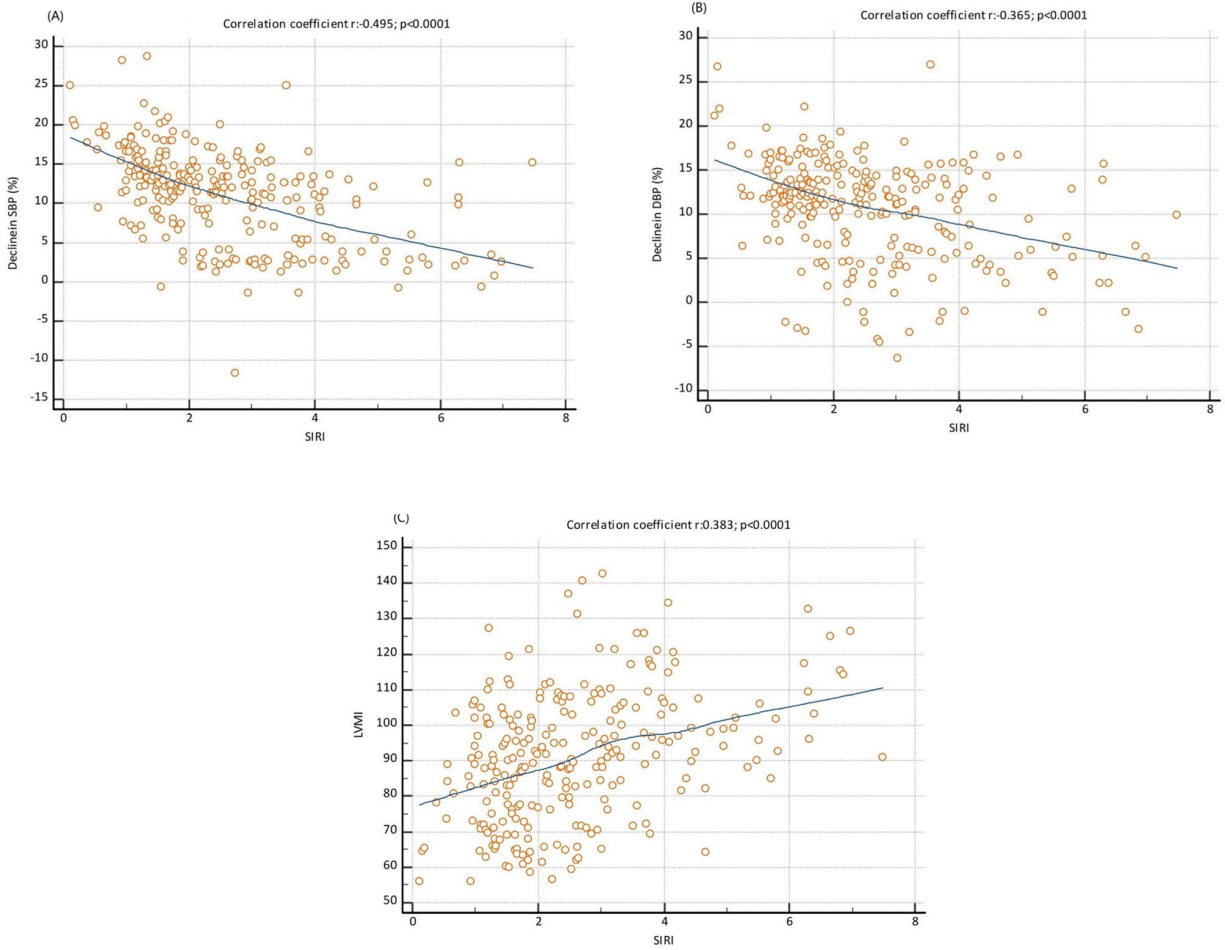
Representation circadian variation of SBP (**A**), DBP (**B**) during the day, and the LVMI (**C**) according to the levels of SIRI in scatter diagram. *SBP* Systolic Blood Pressure, *DBP* Diastolic Blood Pressure, *LVMI* Left Ventricular Mass Index, *SIRI* Systemic Inflammatory Response Index

When comparing echocardiography results of the two groups, patients in the NDHT group were found to have higher IVSd, LVPWd, LVDd, LVMI, and LAd values, and the LVEFs of both groups were found to be similar (Table 1). While the NDHT ratio was similar in both sexes (Fig. 1A), no difference was found in SIRI levels between men and women (Fig. 1B). In the Spearman Rho Correlation Coefficient analysis, a strong and negative correlation was shown between SIRI value and the daily circadian change ratio of SBP and DBP, while a positive and significant correlation was found between SIRI and LVMI (respectively, Fig. 2A–C). The existence of linear relationship between continuous independent variables and dependent variables was investigated by the Box-Tidwell method. As a result of this test, collinearity was determined between platelet and PLR, neutrophil and NLR, and monocyte and MHR. Thus, neutrophil, monocyte, platelet variables were not added to the multiple regression analysis. Multivariate logistic regression analysis revealed that SIRI, HDL-C, and LVMI were independent and significant predictor factors for NDHT (Table 2).

**Table 2:** Effects of various variables on non-dipper hypertension in univariate and multivariate logistic regression analyses

Variables	Univariate analysis		Multivariate analysis	
	OR (95% CI)	*P- value*	OR (95% CI)	*P- value*
LAd	4.704 (1.809–12.236)	0.001	1.923 (0.641–5.773)	0.244
SIRI	1.909 (1.533–2.378)	< 0.001	1.749 (1.076–2.843)	0.024
CRP	1.625 (1.065–2.480)	0.024	1.386 (0.846–2.271)	0.196
NLR	1.408 (1.193–1.661)	< 0.001	0.848 (0.569–1.264)	0.418
MHR	1.045 (1.017–1.074)	0.002	0.986 (0.936–1.038)	0.589
LVMI	1.036 (1.020–1.053)	< 0.001	1.018 (1.000–1.037)	0.046
PLR	1.007 (1.003–1.011)	< 0.001	1.003 (0.998–1.007)	0.293
HDL-C	0.927 (0.886–0.971)	0.001	1.070 (1.008–1.135)	0.027
Platelet	1.002 (0.999–1.005)	0.134	–	
Lymphocyte	0.463 (0.303–0.708)	< 0.001	–	
Neutrophil	1.162 (1.032–1.307)	0.013	–	
Monocyte	2.476 (1.190–5.153)	0.015	–	

*HDL-C* High-Density Lipoprotein Cholesterol, *CRP* C-Reactive Protein, *NLR* Neutrophil/Lymphocyte Ratio, *PLR* Platelet/Lymphocyte Ratio, *SIRI* Systemic İnflammatory Response İndex, *MHR* Monocyte to HDL-C Ratio, *LAd* Left Atrium Diameter, *LVMI* Left Ventricular Mass İndex

**Fig. 3:**
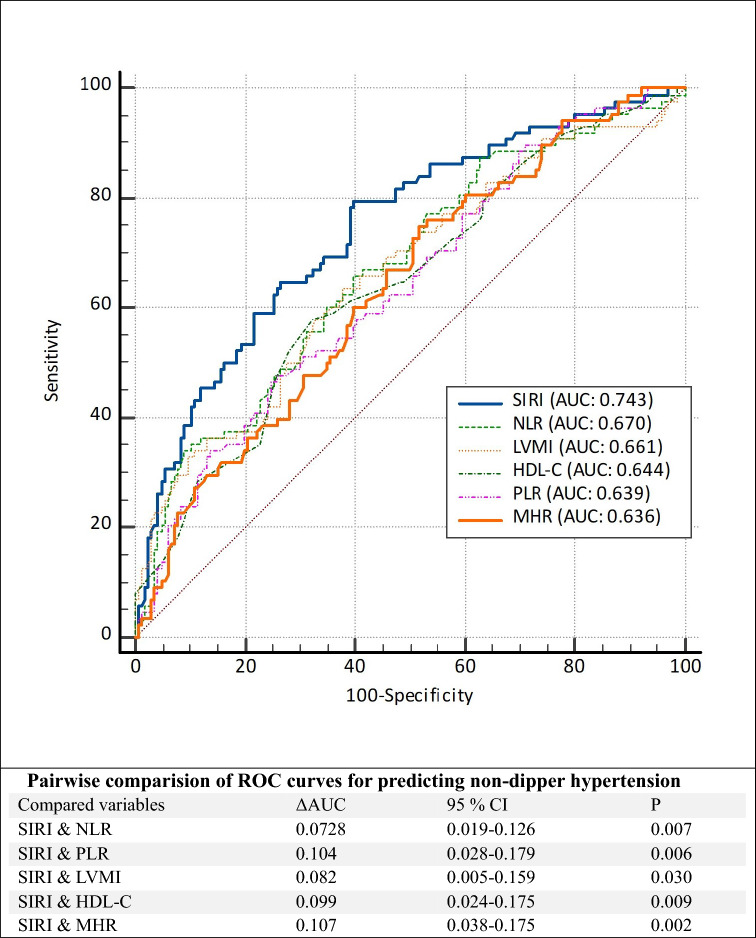
Receiver operating characteristic (ROC) curves of the SIRI, NLR, LVMI, HDL-C, PLR and MHR for predicting an non-dipper hypertension in newly diagnosed hypertensive patients. (*AUC* Area Under The Curve, *CI* Confidence Interval, *LVMI* Left Ventricular Mass Index, *HDL-C* High-Density Lipoprotein Cholesterol, *SIRI* Systemic Inflammatory Response Index, *NLR* Neutrophil/Lymphocyte Ratio, *PLR* Platelet/Lymphocyte Ratio, *MHR* Monocyte to HDL-C Ratio)

ROC curve analysis determined that the optimal SIRI cutoff value for predicting NDHT diagnosis was 2.41 (sensitivity 69.3%, specificity 64.5%, area under the receiver operating characteristic curve, 0.743; *p* < 0.001). ROC curve analysis results indicated that, SIRI demonstrated a superior discriminative capacity for predicting NDHT compared with MHR, NLR, PLR, HDL-C, and LVMI when their AUC values were compared (Fig. 3).

## Discussion

Hypertension is a preventable cause of cardiovascular disease and all-cause mortality [16–18]. Today, significant progress has been made in understanding the epidemiology, pathophysiology and associated diseases of hypertension, and there is strong evidence that early hypertension treatment will significantly reduce morbidity and mortality [15]. Therefore, it is highly important to diagnose and treat hypertension in a timely manner. Blood pressure is generally 10–20% lower at night than during the day. This decrease is not observed in some patients and this condition is known as NDHT. The NDHT is associated with environmental factors, obstructive sleep apnea syndrome, obesity, high salt intake in salt-sensitive patients, orthostatic hypotension, autonomic dysfunction, chronic kidney disease (CKD), diabetes and advanced age [19]. The NDHT is an important risk factor for end-organ damage, and its early detection is critically important. ABPM is currently considered the gold standard diagnostic method for NDHT diagnosis. However, it is also true that ABPM is costly and not an easily accessible test. Therefore, in recent years, alternative markers have been sought for NDHT diagnosis. SIRI is a cheap and easily accessible index obtained from peripheral blood analysis and its prognostic value has been shown in CAD [11] and HF [12]. This study also showed that SIRI is an independent and important predictor factor for NDHT. In addition, this study also revealed that SIRI has a higher predictive power for NDHT than NLP, PLR, and MHR.

Inflammation plays a key role in the pathogenesis of hypertension and hypertension-related complications [20]. Likewise, there are several studies reporting the relationship between NDHT and inflammation. Thus, the relationship between NDHT and inflammation has become a subject of great interest in recent years. In an experimental study carried out in mice, it was revealed that the number of neutrophils was high in the period before hypertension developed [21]. Again, in a study performed by Tatsukawa et al., it was reported that increased neutrophil count was related to the incidence of hypertension in Japanese women [22]. In an animal study, Barhoumi et al. showed that the lymphocyte subset involved in the regulation of T lymphocytes suppressed the level of angiotensin II, which is involved in blood pressure elevation and vascular damage [23]. In this study, in parallel with these findings, neutrophil levels were found to be high in NDHT patients, while lymphocyte levels were found to be low.

According to an earlier research, people with NDHT experience more end-organ damage than people with DHT. Recent data show that norepinephrine levels are higher in individuals with NDHT than in individuals with DHT at night. It is thought that this situation may lead to the development of NDHT by increasing vascular resistance [24]. Additionally, it is thought that high nighttime blood pressure increases the release of proinflammatory cytokines, leading to endothelial damage [25]. Consistent with these observations, studies have demonstrated that NDHT patients have reduced levels of endothelial progenitor cells, which are crucial for endothelial hemostasis and vascular regeneration [26]. As it has been established that hypertension and inflammation are related to each other, it is crucial to carry out research on the relationship between hypertension and indices derived from blood cells taken from peripheral blood. Indeed, a recent study demonstrated that NLR, MHR and PLR are independent predictors for NDHT [8, 9]. Similarly, the current study found that patients with NDHT had higher PLR, MHR and NLR levels compared with those with DHT. SIRI, which includes three parameters—neutrophils, monocytes, and lymphocytes—derived from peripheral blood samples, has been shown to be a good marker for predicting prognosis in cancer, CAD, and HF [10]. Our current study has also revealed that SIRI is an independent factor for predicting NDHT. Another significant point of our study is the pairwise analysis showing SIRI’s effect in predicting NDHT is stronger than that of NLR, MHR and PLR. This could be interpreted as SIRI including three blood parameters instead of two, unlike the other two indices. Additionally, the larger number of patients in our study could have contributed to a clearer demonstration of these factors’ effects.

In this study, the NDHT rate was similar in both sexes. In addition, the SIRI level was found to be higher in women, although not significant. The prevalence of hypertension is as high as 19% in premenopausal women, 44% in perimenopausal women, and 75% in postmenopausal women aged 65–74 years old [27]. Interestingly, data obtained in recent years show that the age group in which the prevalence of hypertension has increased the most is women aged 45–54 years old (perimenopausal period) [28]. It is known that the frequency of hypertension in premenopausal women is less than in men. This is attributed to the high estrogen level and low blood volume in women [27]. In addition, obesity and insulin resistance play an important role in the etiology of hypertension in premenopausal women [29]. Although it varies according to societies and individuals, the average age of menopause 48–52 years old. [30]. In our study, the average age of women was found to be around 51 years old, and this age group is compatible with the perimenopause age group and coincides with the period when the protective effect of estrogen decreases. The fact that the NDHT rate was found to be similar in both sexes in the study can be interpreted as this situation.

Another parameter, low HDL-C, was shown to be an independent predictor factor for NDHT in our study. This aligns with the results of the study carried out by Selçuk and colleagues [24], who also found that the monocyte/HDL-C ratio was higher in patients with NDHT and that this ratio was an independent factor for NDHT. HDL-C is known to have an inverse effect on oxidized LDL-C, control the release of endothelium adhesion molecules, and prevent the activation of leukocyte subgroups like neutrophils and monocytes. Due to these, HDL-C is thought to contribute to vasodilation by increasing the release of nitric oxide from the endothelium [26, 31].

This study revealed that LVMI could be an independent predictor of NDHT and that it was higher in patients with NDHT. Earlier studies have shown that the prevalence of left ventricular hypertrophy and impaired left ventricular diastolic function is more common in patients with NDHT compared with those with DHT [32–34]. Increased renin–angiotensin–aldosterone activity in NDHT is thought to be a contributing factor to this condition, as it causes diastolic dysfunction and left ventricular remodeling [35]. Additionally, NDHT might lead to left ventricular remodeling independent of daytime BP levels by causing an increase in arterial stiffening [36].

## Limitations

The retrospective nature of the study and the small number of patients can be considered as significant limitations. The inclusion of only newly diagnosed naive hypertensive individuals can also be considered as a limitation. The lack of assessment of serum renin–angiotensin–aldosterone levels, which have been shown in some studies to be an independent risk factor for LVMI, can also be considered a limitation. Additionally, experimental animal studies may be needed to elucidate the mechanistic relationship between SIRI and NDHT.

## Conclusions

NDHT leads to increased inflammation, impairment in endothelial functions, and consequently, increased end-organ damage and adverse cardiovascular events. The best test currently available to demonstrate this circadian variation in BP is ABPM. However, due to the cost and accessibility challenges of this test, its application to all hypertensive individuals is not feasible. Therefore, it is believed that there is a need for cheaper and more accessible tests for this diagnosis. This study has shown that SIRI, calculated from subgroups of white blood cells, is an independent and significant factor for the diagnosis of NDHT. This index, derived from a peripheral blood sample, could guide the identification of NDHT patients in situations where ABPM is not possible. However, we believe that further studies with larger patient volumes are needed to validate its use in routine clinical practice.

## Data Availability

All data generated or analyzed during this study are included in this published article.
